# The single cell transcriptional landscape of esophageal adenocarcinoma and its modulation by neoadjuvant chemotherapy

**DOI:** 10.1186/s12943-022-01666-x

**Published:** 2022-10-17

**Authors:** Wayne Croft, Richard P. T. Evans, Hayden Pearce, Mona Elshafie, Ewen A. Griffiths, Paul Moss

**Affiliations:** 1 Institute of Immunology and Immunotherapy, University of Birmingham, Birmingham, UK; 2 Centre for Computational Biology, University of Birmingham, Birmingham, UK; 3 University Hospitals Foundation Trust, Edgbaston, Birmingham, UK; 4 Institute of Cancer and Genomic Sciences, University of Birmingham, Birmingham, UK

**Keywords:** Esophageal adenocarcinoma, scRNA-Seq, Regulatory T cell, Cancer-associated fibroblast, Plasmacytoid dendritic cell

## Abstract

Immune checkpoint blockade has recently proven effective in subsets of patients with esophageal adenocarcinoma (EAC) but little is known regarding the EAC immune microenvironment. We determined the single cell transcriptional profile of EAC in 8 patients who were treatment-naive (*n* = 4) or had received neoadjuvant chemotherapy (*n* = 4). Analysis of 52,387 cells revealed 10 major cell subsets of tumor, immune and stromal cells. Prior to chemotherapy tumors were heavy infiltrated by T regulatory cells and exhausted effector T cells whilst plasmacytoid dendritic cells were markedly expanded. Two dominant cancer-associated fibroblast populations were also observed whilst endothelial populations were suppressed. Pathological remission following chemotherapy associated with broad reversal of immune abnormalities together with fibroblast transition and an increase in endothelial cells whilst a chemoresistant epithelial stem cell population correlated with poor response. These findings reveal features that underlie and limit the response to current immunotherapy and identify a range of novel opportunities for targeted therapy.

**Supplementary Information:**

The online version contains supplementary material available at 10.1186/s12943-022-01666-x.

## Background

Over 570,000 new cases of esophageal adenocarcinoma (EAC) are diagnosed annually and EC remains a tumor of unmet need with high rates of morbidity and mortality and nearly 510,000 annual deaths [1–3]. EC comprises two major histological subtypes, adenocarcinoma or squamous cell carcinoma, with distinct etiological factors and management pathways.

Risk factors for esophageal adenocarcinoma (EAC) include high body mass index and gastrointestinal reflux disease and its incidence is rising markedly in many countries. Many cases develop from Barrett’s esophagus which originates from transformation of gastric cardia through c-MYC and HNF4A-driven transcriptional programming [4]. Surgical resection (esophagectomy) may be curative for some patients without metastatic disease and is usually preceded by administration of chemotherapy or chemoradiotherapy as a ‘neoadjuvant’ treatment [5, 6]. However, patients with metastatic disease are not considered eligible for this approach and the 5-year survival for those who do undergo neoadjuvant chemoradiotherapy and surgery remains below 50% [7].

As such there is considerable interest in the potential utility of immunotherapy regimens to improve EAC clinical outcome. The CheckMate 577 trial demonstrated that adjuvant PD-1 blockade increased disease-free survival from 11 to 24 months in patients with EAC following neoadjuvant chemoradiotherapy and resection [8, 9]. Immune checkpoint inhibition (ICI) has also proven of value in the treatment of advanced squamous cell tumors [10, 11]. Whilst these findings show the potential power of harnessing the immune system in the treatment of esophageal cancer, clinical responses remain suboptimal. To develop more effective and targeted immunotherapy regimens it is now critical to develop a comprehensive understanding of the immune response within the tumor microenvironment in patients with EAC.

scRNA-Seq analyses have transformed understanding of the complexity and heterogeneity of the tumor microenvironment. Recent interrogation of the scRNA-Seq landscape of squamous cell EC has revealed a complex microenvironment with many features of immune suppression including accumulation of proliferative and exhausted CD8+ T cells [12, 13]. SMART-Seq2 analysis of ~ 200 cells from two patients with EAC focussed on tumor cells and identified cellular heterogeneity [14].

scRNA-Seq assessment is particularly powerful when applied to samples from clinical pathways that allow correlation of cellular features with disease progression and treatment. This can be used to assess which cellular subtypes associate with treatment response and so help to guide the introduction of novel therapies. We undertook scRNA-Seq analyses of the EAC tumors in patients with both primary disease or those who had undergone neoadjuvant chemotherapy (NAC). Furthermore, these findings were correlated with pathological response to NAC. We observe that EAC elicits a strong T cell immune response, whose efficacy is likely limited by exhaustion, extrinsic cellular regulation and impaired antigen presentation. A range of discrete tumor-associated stromal populations are also observed. Many of these profiles are corrected by successful neoadjuvant chemotherapy and indicate a wide range of immunotherapeutic opportunities in EAC.

## Methods

### Sample selection criteria and collection

Eight patients with esophageal adenocarcinoma were recruited at University Hospitals Birmingham NHS Foundation Trust, Queen Elizabeth Hospital (Birmingham, UK) under appropriate ethical approval (HBRC 18-304). Four patients had no prior oncological treatment whilst four had received neoadjuvant chemotherapy with four cycles of FLOT (5-FU, Leucovorin, Oxaliplatin, Docetaxel) [5]. Tissue samples were obtained by endoscopic biopsy under general anaesthetic during routine staging or sampling of esophagectomy resection. Endoscopic biopsies were performed by an esophago-gastric resectional surgeon and sampling of resection specimens was performed by a consultant histopathologist with a specialist interest in esophageal cancer. Adjacent normal esophageal tissue samples (*n* = 2) were obtained from an area of macroscopically normal esophagus 2 cm + distant to the tumor.

The Mandard system was used to assess tumor regression grade following neoadjuvant chemotherapy (Score 1, complete regression; 2, rare residual cancer cells; 3, increase in residual cancer cells with fibrosis still predominant; 4, residual cancer outgrowing fibrosis; 5, absence of regressive change [15].

### Sample processing for scRNA-seq

Tumor tissue was collected in MACS® Tissue Storage Solution (Miltenyi Biotec) and cut into small fragments (< 0.5mm^3^) prior to placement in a gentleMACS™ C Tube (Miltenyi Biotec) containing 5 ml pre-warmed DMEM media supplemented with 10% FCS, 1% Penicillin/Streptomycin (all Life Technologies), 100 μg/ml Primocin (InvivoGen), 1X Gentle Collagenase/Hyaluronidase (STEMCELL™ Technologies), 125 μg/ml Liberase TL (Roche) and 50 U/ml Benzonase® (Sigma-Aldrich). Dissociation of tissue fragments was achieved using a gentleMACS™ dissociator (Human Tumor Programme 1, 2 and 3) with incubation at 37 °C for 30 minutes between each agitation. The single cell suspension was filtered through a 70 μm filter and red blood cells were subsequently lysed. Cells were washed and resuspended in MACS buffer (PBS, 0.5% BSA, 2 mM EDTA) prior to immunostaining and FACS sorting. Single cell suspensions of digested tumor tissue were surface stained with anti-CD45 BV785 (2D1, Biolegend), anti-EpCAM APC (9C4, Biolegend) and anti-Podoplanin AF488 (NC-08, Biolegend) to confirm the presence of immune cells, epithelial cells and fibroblasts, respectively. PI was added prior to sorting to exclude non-viable cells. Sorted cell populations were adjusted to 1 × 10^3^/ml. CD45+ cells were dominant and CD45- cells were therefore enriched separately and recombined with CD45+ cells prior to sequencing.

Samples with > 85% cell viability were processed at the Genomics Birmingham Sequencing Facility (University of Birmingham, UK) for gene expression profiling using the 10X Genomics platform. Around 1.7 × 10^4^ cells per sample were processed using the Chromium Controller (10X Genomics) for a recovery of 1 × 10^4^ cells per sample, and library preparation was performed using the Chromium Single Cell 3′ Library & Gel Bead Kit v2 according to manufacturer’s instructions. Library quantification and quality control was performed using TapeStation (Agilent). Ten thousand cells were obtained for each sample which were then sequenced on an Illumina NextSeq 500 (150 bps, paired-end) at a sequencing depth of ~ 20,000 raw reads/cell.

### Processing scRNA-seq data

Raw sequencing read data were processed using Cell Ranger v5.0.1 [16]. Raw read bcl files were converted to fastq and aligned to the Human reference genome GRCh38 with cellranger mkfastq and cellranger count respectively, giving a matrix representing unique molecular identifiers (UMI’s) per cell barcode per gene. The raw UMI matrices for each sample were processed using R v3.6.2 [17] with the Seurat package v3.2.0 [18]. Matrices were filtered to remove cells with < 500 genes detected, > 3500 genes detected and cells with > 10% of reads mapping to mitochondrial RNA. DoubletFinder was used to identify doublets [19] and these were subsequently removed from further analysis.

Cell cycle score for each cell was calculated with Seurat CellCycleScoring function and the difference between G2M and S phase score quantified. For normalisation, Seurat SCTransform function was applied, regressing out percentage mitochondrial mapping and G2M-S phase cell cycle score difference. Data from all samples was then integrated to combine and account for batch effects using the IntegrateData function following Seurat SCTransfrom integration workflow on the top 8 k most variable genes.

### Unsupervised clustering and cell type annotation

The top 8 k most variably-expressed genes were used for dimensionality reduction, firstly by principal component analysis (PCA) and subsequently by uniform manifold projection (UMAP), selecting PCs 1:20 that explained the majority of the variance observed (assessed by elbow plots). A shared nearest-neighbour graph was constructed in PCA-space using PCs 1:20 with Seurat FindNeighbors function. Clusters are identified within this graph using Seurat FindClusters function, optimizing the modularity with the Louvain algorithm. The resolution parameter to control cluster granularity was automatically selected at 0.7 by iteratively increasing this parameter from 0.6 until the criteria of a minimum of 5 differentially expressed (FDR < 0.05 & min. 2-fold expression difference) cluster marker genes were no longer met. Cluster marker genes were identified with FindAllMarkers function using default parameters.

To annotate clusters with high-level cell type, canonical cell type marker gene expression level was assessed in combination with automated cell type annotation using SingleR v1.0.6 [20] on HPCA and Monaco reference sets. High level cell type markers used to inform annotation were CD3D (T cell), MS4A1 (B cell), IGKC (Plasmablast), EPCAM (Epithelial), MKI67 (Cycling), PECAM1 (Endothelial), DCN (Fibroblast), LYZ (Myeloid), TPSAB1 (Mast). Ambiguous cells that could not be clearly assigned to a high-level cell type were removed from further analysis.

For finer grained analysis within high-level cell types, data were subset on the following groupings for independent analysis of each high-level cell type: Lymphocyte T/NK (clusters T and NK), Lymphocyte B (B and Plasmablast), Stromal (Epithelial, Endothelial, Fibroblast, Myofibroblast), Myeloid, Mast and Cycling. Each subset was split back to the raw per-sample UMI matrix data and SCTransform integration procedure applied as previous. Dimensionality reduction, clustering and cell type annotations were then applied on these subsets as previously described. Clusters were annotated with phenotype and main gene discriminating from other clusters wherever possible. The T/NK group were subset further and re-clustered for the finer grained analysis of T and NK cells independently. The stromal grouping was also further subset for the independent analysis of Fibroblasts and Endothelial/Epithelial cells. At each iteration following subsetting and re-clustering, any cells that were carried over due to previous mis-clustering and hence assigned an incorrect cell type were removed from further analysis. Mis-clustered cells were identified by assessing expression of a panel of high-level cell type markers.

### Cluster proportion comparisons

Samplewise proportions of each cluster were calculated and stratified by pre/post chemotherapy, Mandard score and tissue type. Wilcoxon rank sum test was applied to compare the distributions of cluster proportions observed.

### Signature scoring

Cells were scored for signature gene sets of interest using Seurat AddModuleScore function. This score is calculated as the average expression of the gene set per single cell minus background expression from randomly selected control features with positive scores indicating that the gene module is expressed more highly than expected given the average population expression. T cell relevant signatures included naïve, cytotoxic, exhaustion, T regulatory cells, inhibitory checkpoints, stimulatory checkpoints and a core gene signature for tissue resident memory cells [21]. The distribution of signature scores from tumor sample data post and pre chemotherapy were compared using Wilcoxon rank sum test.

### Differential expression

Genes differentially expressed in post vs pre chemotherapy and tumor vs adjacent normal sample data were identified using findMarkers with MAST option (test.use = “MAST”), which uses a hurdle model tailored to scRNA-seq data. MAST is a two-part GLM that simultaneously models how many cells express the gene by logistic regression and the expression level by Gaussian distribution [22]. Differential expression testing is then done using the likelihood ratio test.

### Gene set enrichments

Enrichment for Hallmark gene sets within clusters was assessed by gene set variation analysis (GSVA) pseudo-bulk data using the R package GSVA [23]. The pseudo-bulk dataset was generated by taking the mean within-cluster expression of each gene.

## Results

### The esophageal adenocarcinoma microenvironment comprises multiple cellular subtypes which are differentially modulated by response to neoadjuvant chemotherapy

Tumor tissue was obtained from 4 treatment-naive patients, 3 at esophagogastroduodenoscopy and 1 after surgical resection, as well as from resection specimens of 4 patients who had received neoadjuvant FLOT chemotherapy. Two patient-matched adjacent normal tissue samples were also obtained, 1 treatment-naïve and 1 post-chemotherapy, such that overall a total of 10 tissue samples were obtained from 8 patients.

**Fig. 1:**
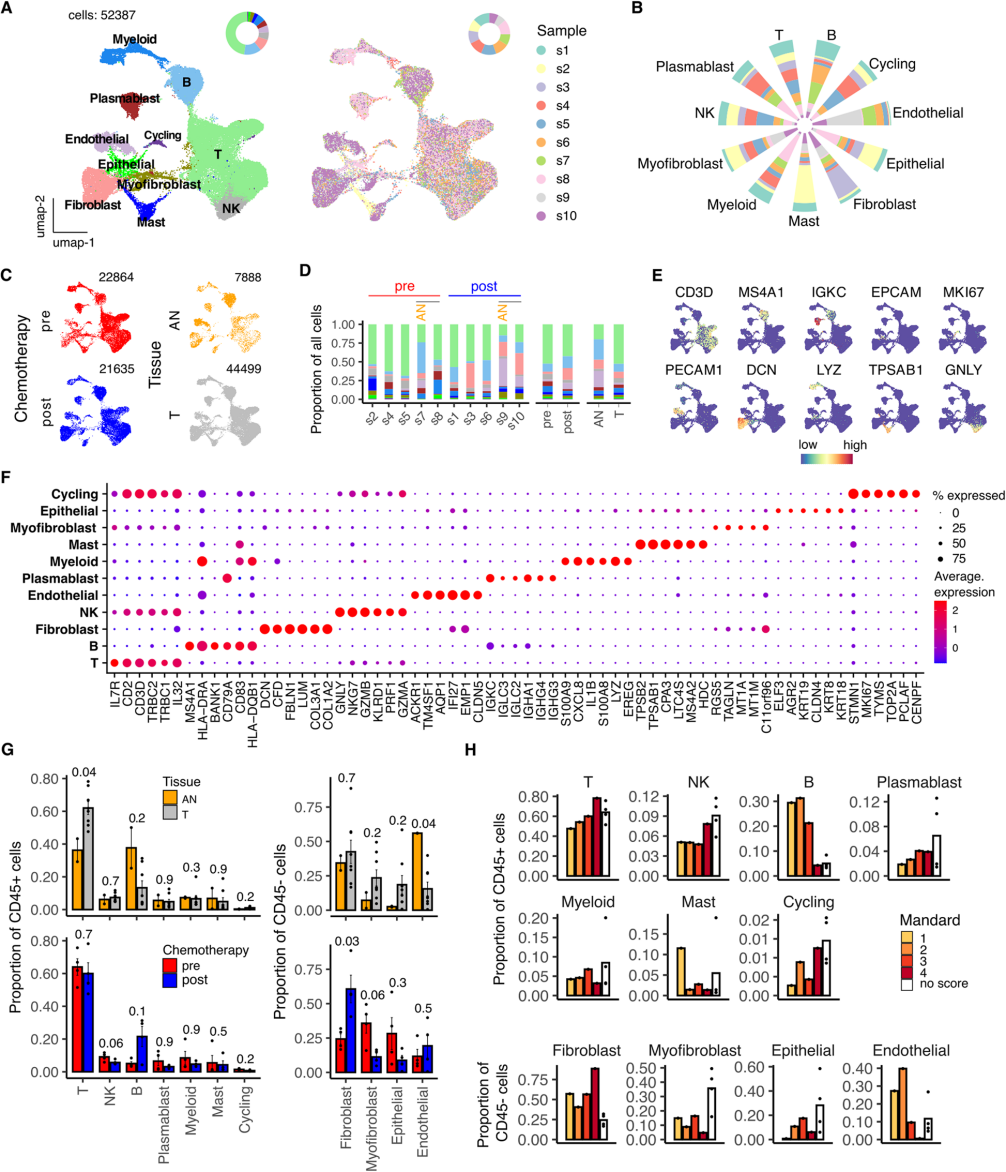
High level cell type ATLAS of Esophageal Adenocarcinoma. **A** UMAP embedding overlaid with unsupervised cluster cell type annotations (left) and sample label (right). **B** Proportional sample contributions to each cell type cluster. **C** UMAP embeddings split by treatment and tissue type. **D** Breakdown of cluster proportions by sample, chemotherapy treatment and tissue type. Grey line indicates matched tumor and Adjacent Normal (AN) samples. **E** UMAP embeddings overlaid with expression of canonical high level cell type marker genes. **F** Average expression profile of top cluster marker genes. Dot size indicates the percentage of the cluster showing expression **G** Comparison by Mann-Whitney test of Adjacent Normal (AN) vs Tumor (T) and pre vs post chemotherapy cluster proportions. **H** Cluster proportions by Mandard scores. Points represent within-sample cluster proportion of total cells and *p* values determined by Mann-Whitney test

Contributions to the total pool of 52,387 single cells studied ranged from 3388 to 6911 cells (6-13%) per sample. Unsupervised clustering was undertaken to determine the profile of the cellular composition within the EAC microenvironment. This identified 10 high-level cell types (T, NK, B, Plasmablast, Myeloid, Mast, Fibroblast, Myofibroblast, Endothelial and Epithelial) together with a small population of cycling lymphocytes (Fig. 1A). Each patient sample contributes to the major cell type clusters identified with fibroblast cells being dominated slightly by s3, Mast cells by s2 and Endothelial cells by s9 (Fig. 1B).

UMAP profiles and cell type contexture were stratified before or after neoadjuvant chemotherapy, and also in comparison of tumor or adjacent normal tissue, to allow transcriptional comparison of homologous populations in different clinical settings (Fig. 1C,D). Expression of canonical cell-type marker genes (Fig. 1E) and automated cell-type classification (Fig. S1B) overlaid reliably on to individual cell types and top cluster marker genes were also defined (Fig. 1F).

The tumor microenvironment (TME) which was dominated by T cells, B cells and fibroblasts with smaller numbers of epithelial cells. Increased proportions of T cells and cycling lymphocytes were seen in tumor compared to normal tissue although endothelial cells were underrepresented (Fig. 1G, H).

Neoadjuvant chemotherapy modulated cellular populations in several ways. Most notable was a reduction in the proportion of NK and proliferative T cells after therapy whilst B cells, endothelial cells and fibroblast populations were increased (Fig. 1G). Response to neoadjuvant chemotherapy was then assessed by Mandard score pathological analysis (Fig. 1H) where 1 refers to complete remission and 5 indicates no pathological response [15]. Unsurprisingly, poor pathological response was associated with increased proportions of epithelial cells, whilst higher proportions of proliferative T cells and NK cells were also retained. In contrast, these cells were markedly suppressed within a complete response and replaced by myofibroblasts, B cells and endothelial cells.

Differential expression analysis was also assessed to identify differentially expressed genes (DEGs) following chemotherapy and comparison to normal tissue (Fig. S1C,D). Chemotherapy induced marked transcriptional change in myofibroblasts (705 DEGs), fibroblast (496 DEGs) and epithelial populations (548 DEGs) (Fig. S1C) whilst myofibroblasts also differed most markedly between normal and tumor tissue (763 DEGs) (Fig. S1D).

### Neoadjuvant chemotherapy reduces T regulatory cells and increases the proportion of effector populations

**Fig. 2:**
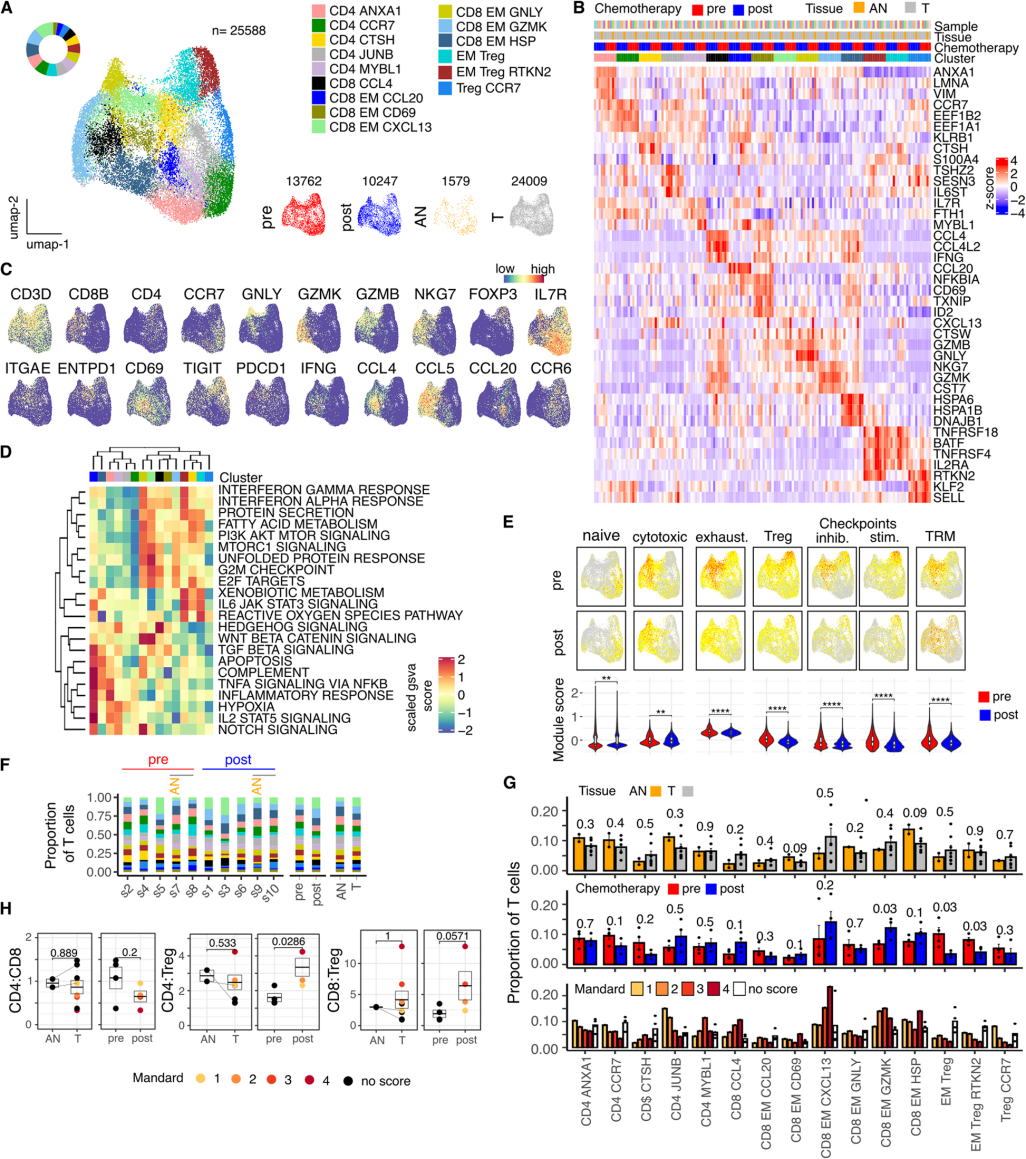
Modulation of T cell contexture, functional signatures and key cell subtype ratios within the tumor microenvironment of Esophageal Adenocarcinoma following NACT. **A** UMAP embedding overlaid with cluster cell type annotations and UMAP embeddings split by treatment and tissue type. **B** Average expression profile of top cluster marker genes. **C** UMAP embeddings overlaid with selected canonical T cell type marker genes. **D** Scaled enrichment score calculated by GSVA for selected MSigDB Hallmark gene sets. **E** UMAP embeddings overlaid with selected signature module scores and distributions of module scores stratified by chemotherapy treatment. **F** Breakdown of cluster proportions by sample, chemotherapy treatment and tissue type. **G** Comparison of Adjacent Normal (AN) vs Tumor (T), pre vs post chemotherapy and Mandard score cluster proportions. Points represent within-sample cluster proportion of total T cells. **H** CD4:CD8, CD4:Treg and CD8:Treg ratios stratified by tissue type and chemotherapy. *P* values determined by Mann-Whitney test

Next we determined the transcriptional profile of each major cell subset within EAC and focused initially on T cells (Fig. 2) which were the dominant lymphocyte population. Unsupervised clustering of 25,588 T cells identified 15 distinct populations of which 5 were CD4+, 7 were CD8+ and 3 represented T regulatory populations (Fig. 2 A,B,C). Marker gene expression (Fig. 2B), automated cell type annotation (Fig. S2B), enrichment of selected hallmark gene sets (Fig. 2D) and signature score distributions (Fig. 2E, S2C) was used to interrogate these in further detail.

Very few naïve T cells were present in tissue and effector responses were dominant (Fig. 2A,F). A CD8+ T population expressing CXCL13, now recognized as a feature of exhaustion in many tumors [24], was increased and associated with poor response to chemotherapy. CD8+ cells expressing CCL4, a chemoattractant for NK cells and monocytes, showed a similar pattern. Moreover, signature scores for cytotoxicity, exhaustion and checkpoint expression were all reduced following chemotherapy.

The CD4:CD8 ratio was 1.1 within tumor tissue prior to chemotherapy but fell to 0.65 following NACT (Fig. 2H). However, chemotherapy markedly reduced the proportion of T regulatory cells within the tumor and the ratio of CD4+ effector:CD4+ regulatory cells increased more than 2-fold from 1.6 to 3.4 (*p* = 0.029) whilst the CD8+:Treg ratio increased from 4 to 6.5 (*p* = 0.057) (Fig. 2H). Effector pools after chemotherapy became enriched in a CD8+ subset enriched for granzyme K expression and CD4+ subset characterized by expression of JUNB (Fig. 2F,G).

### A progenitor NK cell subset is reduced in tumor and increased in patients following chemotherapy

Unsupervised clustering of 1671 NK cells identified five distinct subpopulations including 2 mature CD16+ subsets with predominant expression of CCL3 or FGFBP3. A population expressing *KIT*, the gene encoding CD117 (c-KIT) and characteristic of NK progenitors, was also seen and expressed high levels of IL-7R, IL-4I1 and TNFRSF25 (DR3) (Fig. S3). NK populations within tumor were broadly comparable with normal tissue although the progenitor KIT population was suppressed but increased after chemotherapy in relation to the degree of pathological response, suggesting a potential role in tumor control. In contrast the activated mature populations were reduced following chemotherapy (Fig. S3F). DEG analyses were broadly comparable for each subset within different samples with the GZMK-expressing NK cells being most susceptible to transcriptional modulation post chemotherapy (Fig. S3I).

### The ratio of conventional to plasmacytoid dendritic cells is markedly reduced in tumor but increased following neoadjuvant chemotherapy

**Fig. 3:**
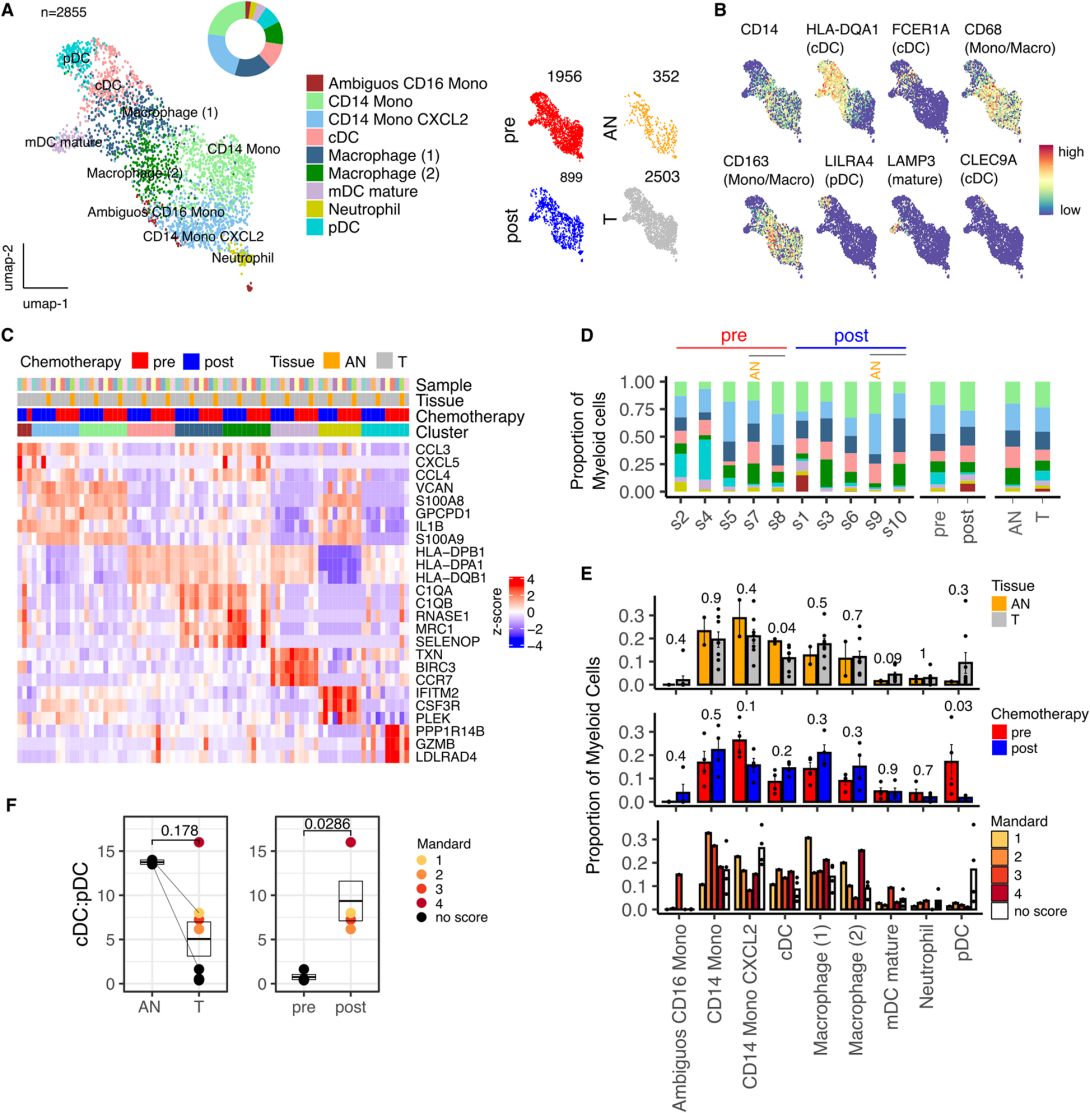
NACT modulation of Myeloid cell contexture identifies shift in cDC:pDC ratio within the tumor microenvironment of Esophageal Adenocarcinoma. **A** UMAP embedding overlaid with cluster cell type annotations and UMAP embeddings split by treatment and tissue type. **B** UMAP embeddings overlaid with expression of canonical Myeloid cell type marker genes. **C** Average expression profile of top cluster marker genes. **D** Breakdown of cluster proportions by sample, chemotherapy treatment and tissue type. **E** Comparison by Mann-Whitney test of Adjacent Normal (AN) vs Tumor (T), pre vs post chemotherapy and Mandard score cluster proportions. Points represent within sample cluster proportion of total sample Myeloid cells. **F** cDC:pDC ratios stratified by tissue type and chemotherapy. *P* values determined by Mann-Whitney test

Two thousand eight hundred fifty-five myeloid cells revealed 9 distinct myeloid cell populations (Fig. 3) comprising 3 dendritic cell populations as well as 2 macrophage, 3 monocytic and a single neutrophil population (Fig. 3, A-C and S4B).

Substantial alteration in the relative distribution of dendritic cells was seen in tumor tissue. In particular, plasmacytoid DC (pDC) were markedly increased whilst conventional DC (cDC) were suppressed (Fig. 3D,E). This ratio was substantially corrected after chemotherapy (Fig. 3F). No additional associations were seen between myeloid subsets and the degree of pathological response during NACT.

### Plasmablast populations are increased in tumor tissue but suppressed following effective pathological response to chemotherapy

**Fig. 4:**
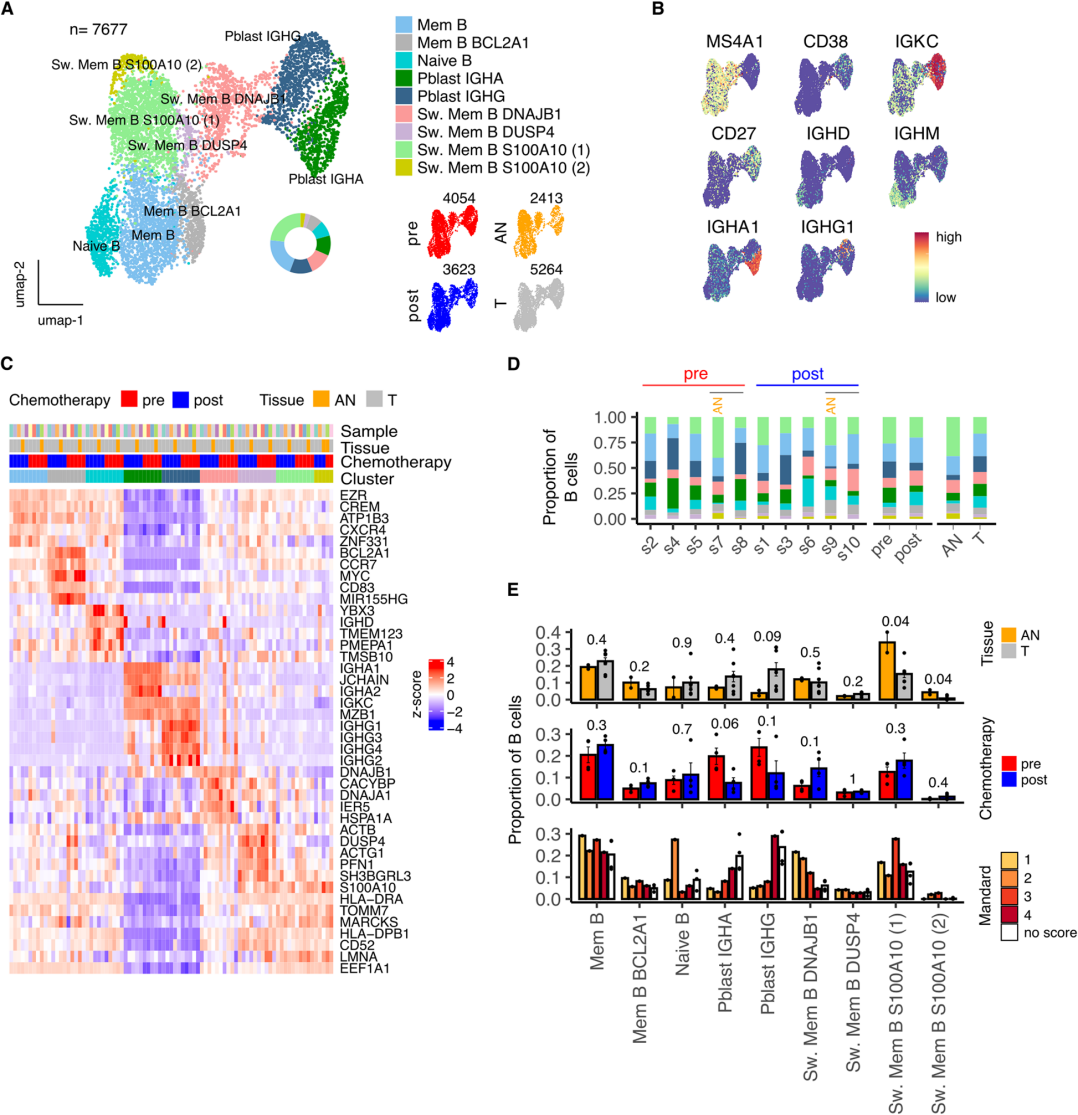
NACT modulation of B cell contexture within the tumor microenvironment of Esophageal Adenocarcinoma. **A** UMAP embedding overlaid with cluster cell type annotations and UMAP embeddings split by treatment and tissue type. **B** UMAP embeddings overlaid with expression of canonical B cell type marker genes. **C** Average expression profile of top cluster marker genes. **D** Breakdown of cluster proportions by sample, chemotherapy treatment and tissue type. **E** Comparison of Adjacent Normal (AN) vs Tumor (T), pre vs post chemotherapy and Mandard score cluster proportions. Points represent within sample cluster proportion of total sample B cells and *p* values determined by Mann-Whitney test

Nine subpopulations of B cells were identified from unsupervised clustering of 7677 cells and comprised 1 naïve, 2 memory and 4 switched memory subsets together with 2 plasmablast populations (Fig. 4A). Expression of canonical B cell subtype markers (Fig. 4B), top cluster markers (Fig. 4C) and automated cell-type annotation (Fig. S4F) confirmed cell type identity.

Switched memory B cells, characterized by expression of S100A10, a negative regulator of Toll-like receptor function, were reduced in tumor tissue whilst plasmablast populations were increased (Fig. 4D, E). This profile was corrected by NACT with the scale of improvement correlating with the quality of pathological response (Fig. 4E). A noteworthy feature was that the DNAJB1+ switched memory subset showed striking transcriptional divergence following chemotherapy, indicating profound cellular plasticity (Fig. S4G).

### Two dominant populations of cancer-associated fibroblasts are present with EAC and suppressed by neoadjuvant chemotherapy

**Fig. 5:**
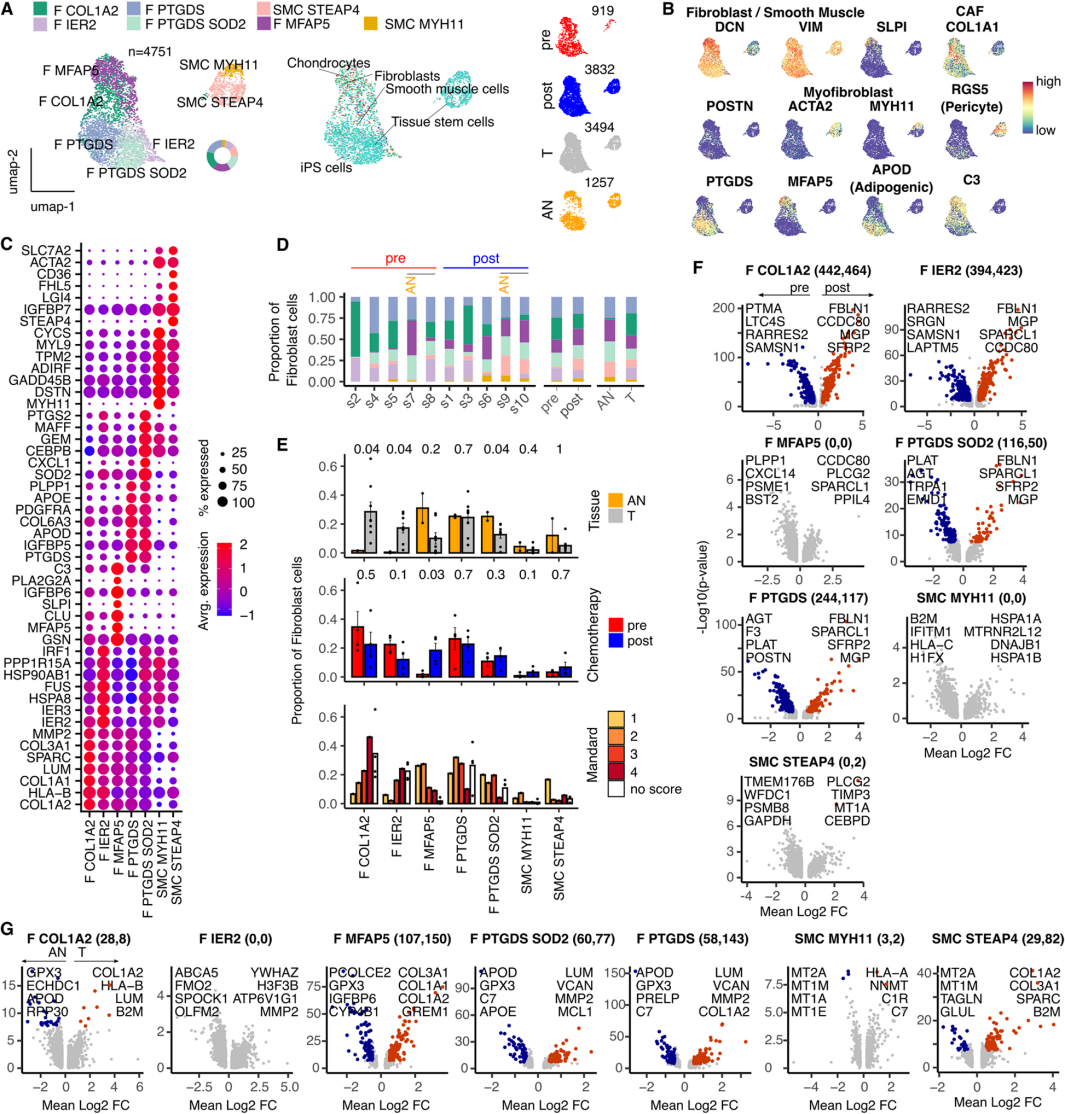
Fibroblast contexture within the tumor microenvironment of Esophageal Adenocarcinoma and gene expression profile changes following NACT. **A** UMAP embedding overlaid with cluster cell type annotations**,** UMAP embeddings split by treatment and tissue type and Automated per-cell annotations of cell type using SingleR with the hpca coarse reference dataset. **B** UMAP embeddings overlaid with selected canonical Fibroblast sub-type marker genes. **C** Average expression profile of top cluster marker genes. Dot size indicates the percentage of the cluster showing expression. **D** Breakdown of cluster proportions by sample, chemotherapy treatment and tissue type. **E** Comparison of Adjacent Normal (AN) vs Tumor (T), pre vs post chemotherapy and Mandard score cluster proportions. Points represent within sample cluster proportion of total sample Fibroblast cells and *p* values determined by Mann-Whitney test. **F** Summary of genes identified as differentially expressed in post vs pre NACT EAC tumor sample data. DEG count noted alongside cluster: (pre expresn=2654sed, post expressed). **G** Summary of genes identified as differentially expressed in EAC Tumor (T) vs Adjacent Normal (AN) sample data. DEG count noted alongside cluster: (AN expressed, T expressed). Coloured points indicate DEGs (BH adjusted *p* < 0.001 and absolute average log2FC > 0.5)

Unsupervised clustering identified 7 distinct fibroblast sub-populations from 4751 cells (Fig. 5A) and these showed considerable variation in relation to tissue sample and chemotherapy. Myofibroblasts (SMC MYH11/STEAP4), adipogenic (PTGDS) and complement expressing (MFAP5) populations were identified via canonical fibroblast subtype marker expression (Fig. 5B) and subsets further characterized by top marker gene expression profile (Fig. 5C) and automated cell type annotations (Fig. 5A).

Two fibroblast subpopulations, characterized by high level expression of COL1A2 and IER2, were markedly increased within the tumor microenvironment and almost completely absent in adjacent normal esophageal tissue (Fig. 5D, E). These were somewhat reduced by chemotherapy although their presence correlated with poor pathological response (Fig. 5E). Both populations displayed striking transcriptional divergence following chemotherapy exposure with 906 and 817 DEGs within the COL1A2 and IER2 populations respectively (Fig. 5F), whilst the MFAP5 fibroblasts showed highest transcriptional difference in tumor vs normal comparisons (Fig. 5G).

In contrast, the residual 5 fibroblast populations, including myofibroblasts, were reduced within tumor tissue but increased following NACT where adipogenic and complement expressing populations increased in proportion to degree of pathological response (Fig. 5E).

### Increased proportions of VEGFA-expressing mast cells in EAC indicate a pro-tumorigenic role

Mast cells are emerging as critical regulators of tumor progression and scRNA-Seq analysis in non-EAC tumors has revealed substantial increase in many tumors [25]. Four mast cell clusters were identified through scRNA-Seq and an additional tumor-associated cluster of 1532 cells was seen in a single donor (s2) (Fig. S5A,B). VEGFA and TNF are key determinants of mast cell activity with pro-tumorigenic or suppressive roles respectively and the VEGFA:TNF ratio acts as a surrogate marker of tumor progression [25].

Most mast cells expressed VEGFA and TGFB, indicating a potential pro-tumoral capacity, whilst TNF expression was low (Fig. S5C). CD69 and CD63 expression was increased, particularly after chemotherapy, and may indicate tissue retention or local activation. Relatively few differences in cluster proportion were observed in relation to tumor microenvironment, chemotherapy or Mandard score (Fig. S5E) although transcriptional activity of most mast cell subtypes was highly modified by chemotherapy (Fig. S5F) and the LMNA expressing Mast subtype was most modulated in tumor compared to adjacent normal tissue (Fig. S5G). These findings indicate a likely pro-tumoral role of mast cells in EAC.

### Epithelial cells are a minority population and selective survival of stem progenitor cells following chemotherapy identifies a range of potential therapeutic targets

**Fig. 6:**
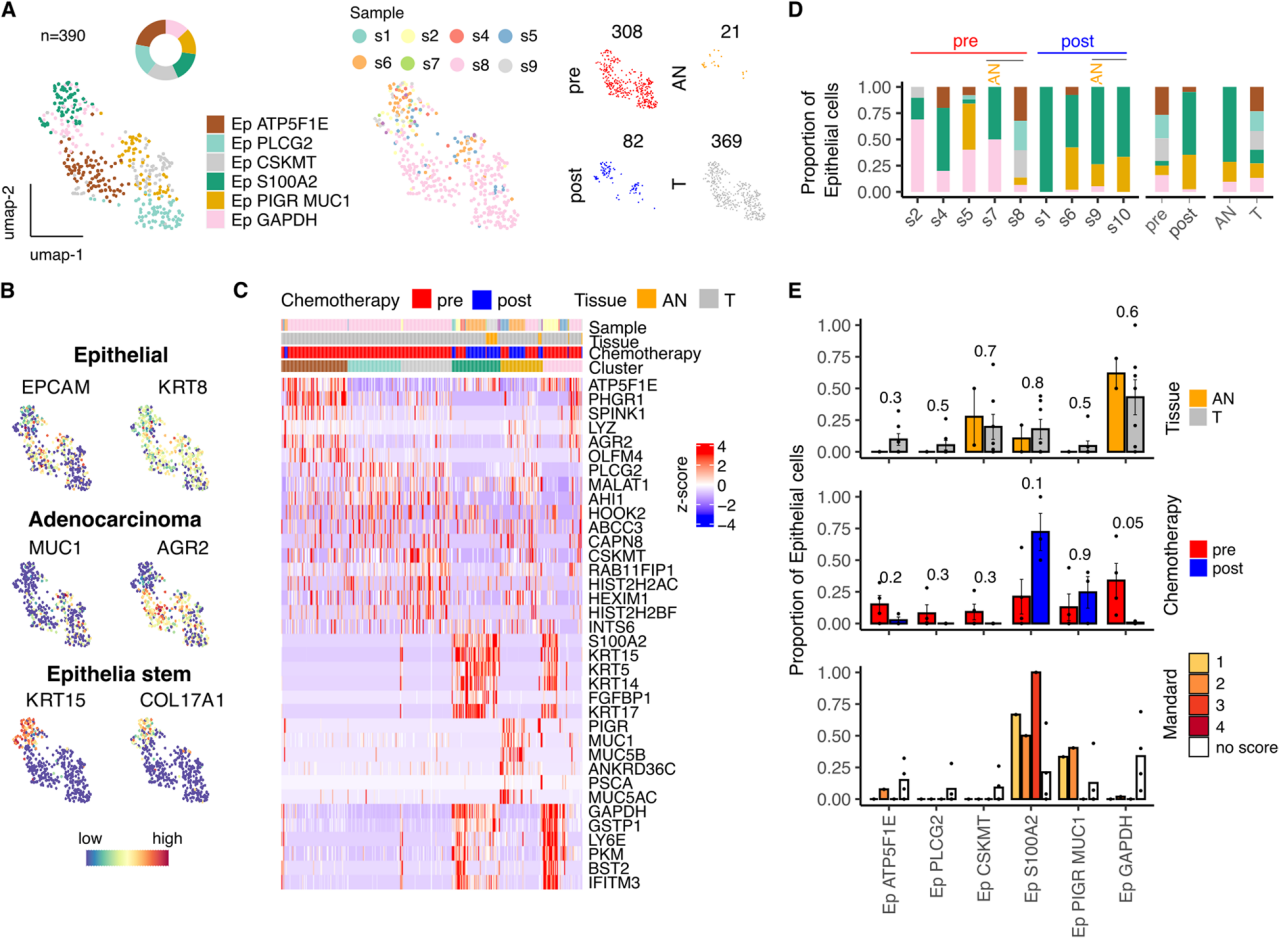
Characterizing Epithelial/Tumor cell subsets within the tumor microenvironment of Esophageal Adenocarcinoma. **A** UMAP embedding overlaid with cluster cell type annotations and UMAP embeddings split by treatment and tissue type. **B** UMAP embeddings overlaid with expression of canonical Epithelial cell type markers. **C** Average expression profile of top cluster marker genes. **D** Breakdown of cluster proportions by sample, chemotherapy treatment and tissue type. **E** Comparison of Adjacent Normal (AN) vs Tumor (T), pre vs post chemotherapy and Mandard score cluster proportions. Points represent within-sample cluster proportion of total sample epithelial cells and *p* values determined by Mann-Whitney test

Unsupervised clustering on 390 epithelial cells identified 6 clusters (Fig. 6) of which 4 were enriched within tumor and are likely dominated by the primary tumor population (Fig. 6A-C). Eleven DEGs were observed between tumor and adjacent normal tissue including S100P, PHGR1 and S100A6 (Fig. S5E). The potential importance of the S100 family of calcium binding proteins was further seen within a subpopulation of tumor-enriched epithelial cells defined by high expression of S100A2, which increased post-chemotherapy and whose expression correlated with poor response (Fig. 6D,E). These cells expressed KRT15 and COL17A1, markers for stem and progenitor esophageal epithelial cells [26, 27] and were notable for their relative chemoresistance suggesting that they may play a potential role in disease relapse (Fig. 6B,C).

### Endothelial cell contexture in the TME of EAC and its modulation by chemotherapy

**Fig. 7:**
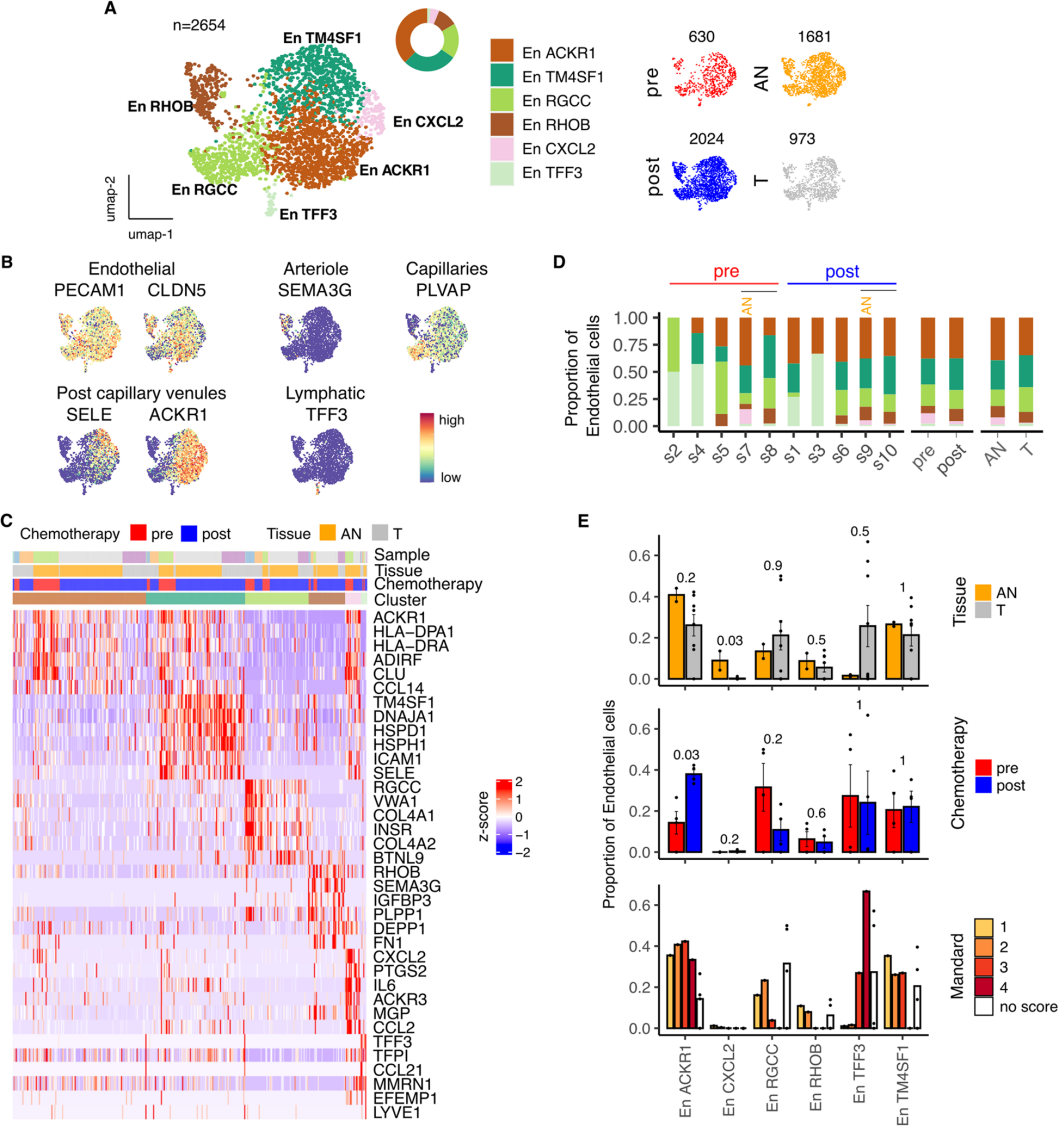
Characterizing Endothelial cells within the tumor microenvironment of Esophageal Adenocarcinoma. **A** UMAP embedding overlaid with cluster cell type annotations and UMAP embeddings split by treatment and tissue type. **B** UMAP embeddings overlaid with expression of canonical Endothelial cell type markers. **C** Per-cell expression profile of top cluster marker genes. **D** Breakdown of cluster proportions by sample, chemotherapy treatment and tissue type. **E** Comparison of Adjacent Normal (AN) vs Tumor (T), pre vs post chemotherapy and Mandard score cluster proportions. Points represent within sample cluster proportion and *p* values determined by Mann-Whitney test

As indicated earlier, the overall proportion of endothelial cells was markedly reduced within the tumour microenvironment. Six subpopulations of endothelial cells were identified by unsupervised clustering of 2654 cells and included distinct arteriole, capillary, venule and lymphatic-type endothelial populations (Fig. 7A-C). The relative proportion of 4 clusters was comparable between tumor and normal tissues and included the dominant capillary, arteriole, post capillary venule and lymphatic vessels. High levels of HLA class II expression were observed in the large post capillary venule cluster (Fig. 7C) indicating an immune surveillance role. The largest endothelial population, defined by expression of the atypical chemokine receptor ACKR1, was significantly increased following chemotherapy (Fig. 7D,E).

A striking reduction in a CXCL2-defined population was seen in tumor endothelial cells (TEC) which showed little recovery after chemotherapy (Fig. 7E). This expressed COX2 and IL-6 and as such would be expected to have an inflammatory role within normal tissue.

In contrast, the proportion of a TFF3-expressing TEC population with features of high endothelial venules was markedly increased in tumour and its retention correlated with poor response to chemotherapy (Fig. 7E). Expression of CCL21, a characteristic marker of HEV, was almost selectively seen in this subset whilst LYVE1 was also expressed (Fig. 7C). DEG analysis after chemotherapy showed only moderate transitions (Fig. S7I) whilst tumor-associated DEGs included ACKR1, RGCC and TM4SF1 (Fig. S7J).

### Proliferative tissue resident T cells are observed only within tumour and become activated by chemotherapy

Finally, expression of cell cycle-associated genes such as *MKi67, TYMS, TOP2A, PCLAF* and CENPF was used to define the presence of cycling cells (Fig. 1E). Of note, this population was seen almost exclusively within tumor where unsupervised clustering identified 4 subsets within 436 cells (Fig. S7A-C) whilst only 10 cycling cells were seen within normal tissue (Fig. S7A).

Cycling cells were predominantly T cells and mostly expressed the tissue residency marker CD103 (*ITGAE*) together with CD39 (*ENTPD1*) which has been associated with tumor specificity (Fig. S7B). Proliferation was broadly equivalent in CD4+ and CD8+ subsets. Chemotherapy reduced the proportion of cycling cells but had no influence on the contexture of the population (Fig. S7A,D). However, substantial differential gene expression was observed including upregulation of MHC class II genes (*HLA-DRB1, HLA-DQB1*) and *ITGB2* (CD18) whilst markers of regulatory function such as GITR (*TNFRSF18*) and IL2RA were reduced (Fig. S7G).

## Discussion

**Fig. 8:**
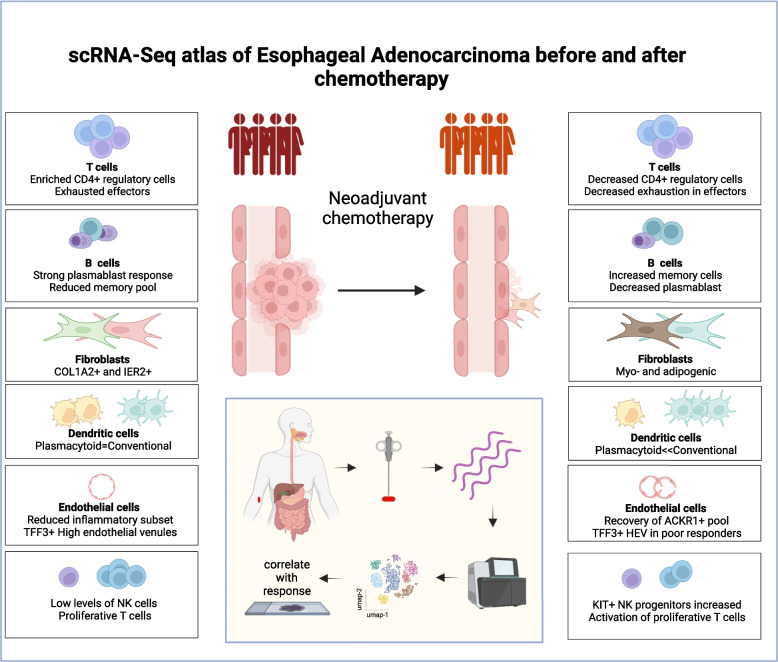
Schematic representation of major findings

Recent studies have demonstrated encouraging but suboptimal clinical responses to immune checkpoint blockade in patients with esophageal adenocarcinoma. As such, detailed assessment of the EAC microenvironment is now required and here we undertook comprehensive scRNA-Seq analyses and interrogated the data in relation to neoadjuvant chemotherapy history and pathological response. UMAP transformations were comparable across donors and samples and allowed direct transcriptional comparisons of cell lineages in relation to disease status. This identified a range of findings that may guide future therapeutic approaches (Fig. 8).

It was noteworthy that epithelial cells were a minority population in both normal and tumor tissue and the microenvironment was dominated by immune and stromal populations. T cells were the majority immune population, a feature also seen in squamous cell esophageal carcinoma [13]. This suggests that esophageal cancer is broadly immunogenic and induces a tumor-specific cytotoxic cellular response in many cases, a concept supported by initial clinical efficacy of PD-1 checkpoint blockade. Intratumoral CD8+ T cells were enriched in CXCL13 and CCL4 expression, mediators of immune cell recruitment, although whether this has a positive or negative influence on tumor growth is unclear. CXCL13 plays a key role in the development of tertiary lymphoid structures through engagement with CXCR5+ follicular helper cells and B cell recruitment, although CD8 + CXCL13+ T cells are observed in many tumors and enriched within exhausted PD-1^high^ subsets [14, 28, 29]. CCL4 attracts innate cells and its serum level is increased in EAC where it correlates with both the degree of lymphocytic infiltrate and superior clinical outcome [30]. Important modifications of the T cell repertoire were seen after chemotherapy with a marked reduction in T regulatory populations together with relative enhancement of effector pools. This underpins the importance of chemotherapy in overcoming cellular suppression of tumor-specific immune responses. CD8+ T cells expressing granzyme K were increased after chemotherapy, consistent with the role of this population as a pre-dysfunctional CD8+ subset [24], and potentially explaining the synergy of preoperative neoadjuvant therapy with subsequent checkpoint blockade. In addition, the transcriptional exhaustion signature of CD8+ subsets was reduced after chemotherapy, further supporting a rebalancing of effector responses as a potential mechanism underlying the efficacy of NACT. The potential importance of T cell immunity in EAC was further revealed by the finding of proliferative cells within tumour that expressed CD39+, a marker of tumour-specific response [31]. Furthermore, chemotherapy acted to increase HLA-DR+ activation status of this pool whilst suppressing markers such as GITR associated with a regulatory phenotype and further reinforcing the concept that chemotherapy acts to increase the inflammatory balance of the microenvironment within tumors.

NK subpopulations revealed a KIT+IL-7R+ progenitor subset that was suppressed within tumor but increased in patients who obtained good pathological response to NACT. Of note, these cells expressed high levels of IL4I1, an enzyme which accelerates the expansion of CD8+ T cells [32], and as such this subset may help to support tumor-specific surveillance.

Profound alterations in the relative distribution of dendritic cells were seen within the tumor microenvironment. In particular, the proportion of plasmacytoid DC was substantially increased whilst conventional DC, which play a major role in driving inflammatory Th1 responses and were markedly dominant in adjacent normal tissue, were reduced. As such the cDC:pDC ratio of 14 in normal tissue was reduced to only 0.75 within tumor tissue, recovering somewhat to 9.4 after chemotherapy. Although plasmacytoid DC normally exhibit robust IFN-α production following TLR-mediated activation they can lose this function with chronic activation and contribute to an immunosuppressive tumor microenvironment [33]. Indeed, they have been suggested a potential target for therapy in gastric cancer [34] and these data indicate that this may also be the case for EAC where it was also noteworthy that they were highly sensitive to chemotherapy.

The potential importance of localized tertiary lymphoid structures in the immune control of tumors is receiving great interest at the current time. EAC tumors contained large populations of both IgG and IgA plasmablasts which were partially suppressed by chemotherapy although retained within tumors with poor pathological response. This indicates that EAC induces an ongoing antibody response, although the functional importance of this is unclear. Indeed, switched memory B cell populations were reduced within the tumor microenvironment and may indicate poor functional maturation of plasmablast populations. Peritumoral B cells can drive angiogenic responses in squamous esophageal tumors but no increase in HMGB1 expression was seen within EAC [35].

Stromal cells play an important role in tumor progression and there is considerable interest in identification and targeting of cancer-associated fibroblasts [36]. Profound alterations in fibroblast subpopulations were seen in EAC with a striking increase in two subsets, defined by expression of COL1A2 and IER2, which were almost completely absent in adjacent normal tissue. COL1A2 promotes epithelia-to-mesenchymal transition [37] whilst its knockdown suppresses proliferation and metastasis of EAC cell lines [38]. These populations may therefore represent valuable therapeutic targets whilst additional fibroblast populations, including myofibroblasts and adipogenic subsets, were seen to increase after successful chemotherapy. Differential gene expression following chemotherapy was most pronounced within fibroblast populations and reflects their high level of tissue plasticity.

Increased proportions of mast cells are seen in many cancer subtypes and are believed to be important in tumorigenesis [39]. scRNA-Seq analyses in non-EAC tumors has identified the VEGFA:TNF ratio as an important determinant of tumor progression and here we also observed dominant VEGFA expression, indicating a likely pro-tumoral role for mast cells in EAC, although trials of VEGF inhibitors in this setting have been largely disappointing [40]. No major changes in mast cell profile were seen after treatment although a population characterized by heat shock protein expression was moderately increased.

A notable opportunity with scRNA-Seq is to assess the unique transcriptional features of primary tumor cells. Increased expression of known EAC-associated genes such as MUC1 and AGR2 [41, 42] was observed within the tumour. Furthermore, expression of the OLFM4 gene associated with nodal metastasis [43] was focused within a single tumor-associated cluster and indicates the potential of single cell analysis to define clusters associated with specific clinical features. Several genes within the S100 family were increased in tumor cells including a tumor-enriched cluster expressing S100A2 together with KRT15 and COL17A1 which are notable as markers of a quiescent stem/progenitor cell population in the most basal layer of the human esophagus [26, 27]. The observation that this population increased after chemotherapy and was associated with poor clinical response suggests that these may have a potential role in tumor resistance and could represent an important therapeutic target (Fig. 6F). A total of 95 genes were differentially expressed in epithelial cells following chemotherapy including upregulation of IGFBP2 which is associated with primary EAC chemoresistance [42, 44].

Tumor endothelial cells (TEC) are critical for growth and metastasis but comprise a heterogeneous repertoire with inherent plasticity for differentiation [45]. Interestingly, endothelial cells were the strongest cellular correlate of clinical state, being markedly suppressed within tumors but recovering strongly in those patients who obtained a good pathological response to chemotherapy. Endothelial cells characterized by expression of the atypical chemokine receptor ACKR1 were increased after NEC which is noteworthy given their association with prevention of tumor progression in other settings [46]. In contrast, a TEC subpopulation expressing CXCL2, IL-6 and ACKR3 was markedly suppressed in tumor with only marginal increase after chemotherapy. ACKR3 has a primary role as mediator of CXCL12 activity and has pleiotropic roles in tumor development [46]. Interestingly many TEC subsets upregulate the pro-angiogenic protein perlecan (*HSPG2*) [47] although clinical responses following anti-angiogenesis therapy in EAC are modest and were associated with poor tissue healing [40].

## Conclusions

In conclusion, we show that EAC tumors direct the development of a complex local microenvironment that drives a strong T cell immune response whose efficacy is limited by effector cell exhaustion and expansion of regulatory subsets. This associates with expansion of plasmacytoid dendritic cells and cancer-associated fibroblasts, whilst endothelial cells are markedly suppressed. Effective neoadjuvant chemotherapy was seen to reverse these changes with suppression of T regulatory pools, correction of dendritic subsets, transition of the fibroblast populations and expansion of endothelial cell subsets. This leads to development of pre-dysfunctional effector T cells and a robust B cell expansion. These findings may help to guide the introduction of novel immunotherapeutic treatments for patients with EAC.

## Supplementary Information

**Additional file 1: Supplementary Fig. S1.** Quality metric distributions, SingleR annotations and differential expression analysis of high level cell types. **Supplementary Fig. S2.** Quality metric distributions, SingleR annotations and differential expression analysis of T cells. **Supplementary Fig. S3.** NACT modulation of NK cell contexture within the tumour microenvironment of Esophageal Adenocarcinoma. **Supplementary Fig. S4.** Quality metric distributions, SingleR annotations and differential expression analysis of Myeloid and B cells. **Supplementary Fig. S5.** Quality metric distributions, SingleR annotations and differential expression analysis of Mast cells. **Supplementary Fig. S6.** Quality metric distributions, SingleR annotations and differential expression analysis of Epithelial and Endothelial cells. **Supplementary Fig. S7.** Quality metric distributions, SingleR annotations and differential expression analysis of Cycling cells.

## Data Availability

The datasets used and/or analysed during the current study are available from the corresponding author on reasonable request.

## Abbreviations

EAC: esophageal adenocarcinoma
ICI: Immune checkpoint inhibition
UMAP: Uniform Manifold Approximation and Projection
NAC: neoadjuvant chemotherapy
TME: tumor microenvironment
pDC: plasmacytoid DC
NK: Natural Killer Cell
TEC: tumor endothelial cells
