# Supplementary material for: The single cell transcriptional landscape of esophageal adenocarcinoma and its modulation by neoadjuvant chemotherapy
Source: Mol Cancer. 2022 Oct 17;21:200. doi: 10.1186/s12943-022-01666-x (PMC9575245; doi:10.1186/s12943-022-01666-x)
Supplement: Supplementary file 1 — Additional file 1: Supplementary Fig. S1. Quality metric distributions, SingleR annotations and differential expression analysis of high level cell types. Supplementary Fig. S2. Quality metric distributions, SingleR annotations and differential expression analysis of T cells. Supplementary Fig. S3. NACT modulation of NK cell contexture within the tumour microenvironment of Esophageal Adenocarcinoma. Supplementary Fig. S4. Quality metric distributions, SingleR annotations and differential expression analysis of Myeloid and B cells. Supplementary Fig. S5. Quality metric distributions, SingleR annotations and differential expression analysis of Mast cells. Supplementary Fig. S6. Quality metric distributions, SingleR annotations and differential expression analysis of Epithelial and Endothelial cells. Supplementary Fig. S7. Quality metric distributions, SingleR annotations and differential expression analysis of Cycling cells. [file 12943_2022_1666_MOESM1_ESM.docx]

**Supplementary Information**


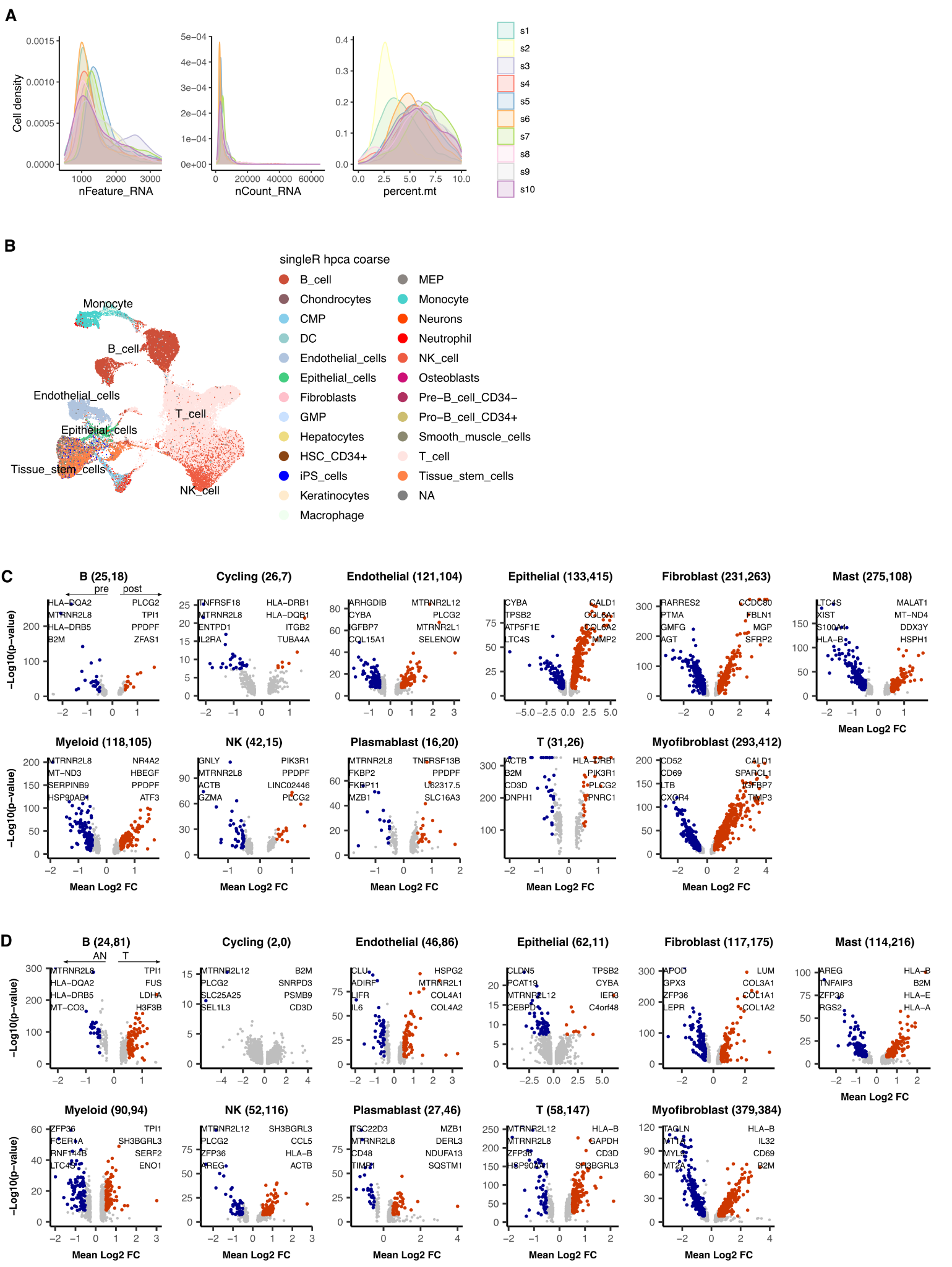


**Supplementary Figure S1. Quality metric distributions, SingleR annotations and differential expression analysis of high level cell types.** A, Samplewise distributions of Feature counts (nFeature_RNA), UMI counts (nCount_RNA) and % expression of Mitochondrial genes (percent.mt). B, Automated per-cell annotations of high level cell types using SingleR with the hpca coarse reference dataset. C, Summary of genes identified as differentially expressed in post vs pre NACT EAC tumor sample data. DEG count noted alongside cluster: (pre expressed, post expressed). D, Summary of genes identified as differentially expressed in EAC Tumor (T) vs Adjacent Normal (AN) sample data. DEG count noted alongside cluster: (AN expressed, T expressed) Coloured points indicate DEGs (BH adjusted p<0.001 and absolute average log2FC > 0.5).

**
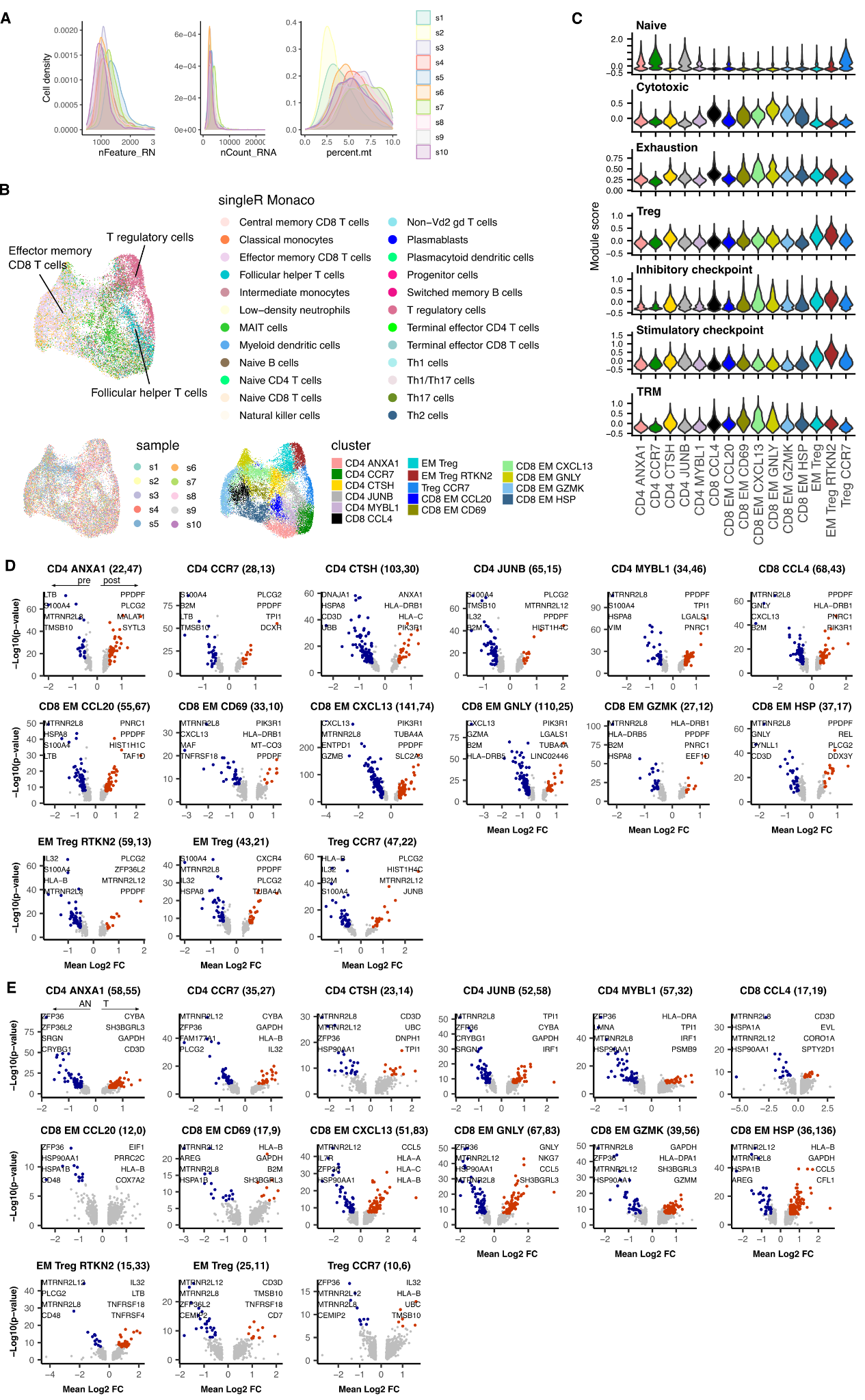
**

**Supplementary Figure S2. Quality metric distributions, SingleR annotations and differential expression analysis of T cells.** A, Samplewise distributions of Feature counts (nFeature_RNA), UMI counts (nCount_RNA) and % expression of Mitochondrial genes (percent.mt). B, Automated per-cell annotations of high level cell types using SingleR with the Monaco reference dataset; UMAP embedding overlaid with sample number; UMAP embedding overlaid with unsupervised clustering cell type annotation. C, Cluster-wise distributions of selected signature module scores. D, Summary of genes identified as differentially expressed in post vs pre NACT EAC tumor sample data. DEG count noted alongside cluster: (pre expressed, post expressed). E, Summary of genes identified as differentially expressed in EAC Tumor (T) vs Adjacent Normal (AN) sample data. DEG count noted alongside cluster: (AN expressed, T expressed) Coloured points indicate DEGs (BH adjusted p<0.001 and absolute average log2FC > 0.5).


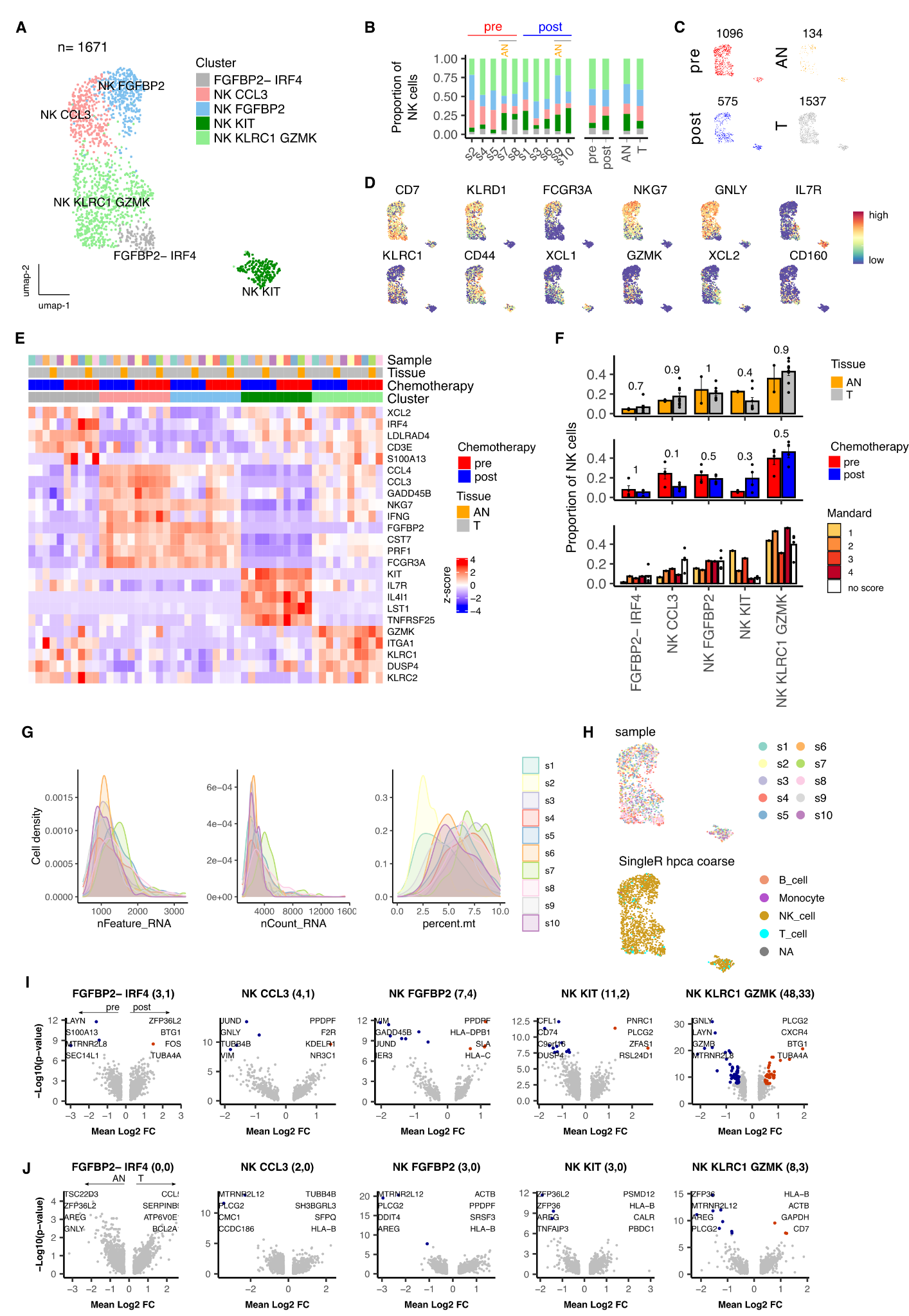


**Supplementary Figure S3. NACT modulation of NK cell contexture within the tumour microenvironment of Esophageal Adenocarcinoma. A.** UMAP embedding overlaid with cluster cell type annotations. **B.** Breakdown of cluster proportions by sample, chemotherapy treatment and tissue type. **C.** UMAP embeddings split by treatment and tissue type. **D.** UMAP embeddings overlaid with expression of canonical NK cell type marker genes. **E.** Average expression profile of top cluster marker genes. **F.** Comparison of Adjacent Normal (AN) vs Tumor (T), pre vs post chemotherapy and Mandard score cluster proportions. Points represent within sample cluster proportion of total sample NK cells and p values determined by Mann-Whitney test. G, Samplewise distributions of Feature counts (nFeature_RNA), UMI counts (nCount_RNA) and % expression of Mitochondrial genes (percent.mt). H, Automated per-cell annotations of high level cell types using SingleR with the hpca coarse reference dataset. I, Summary of genes identified as differentially expressed in post vs pre NACT EAC tumor sample data. DEG count noted alongside cluster: (pre expressed, post expressed). J, Summary of genes identified as differentially expressed in EAC Tumor (T) vs Adjacent Normal (AN) sample data. DEG count noted alongside cluster: (AN expressed, T expressed) Coloured points indicate DEGs (BH adjusted p<0.001 and absolute average log2FC > 0.5).


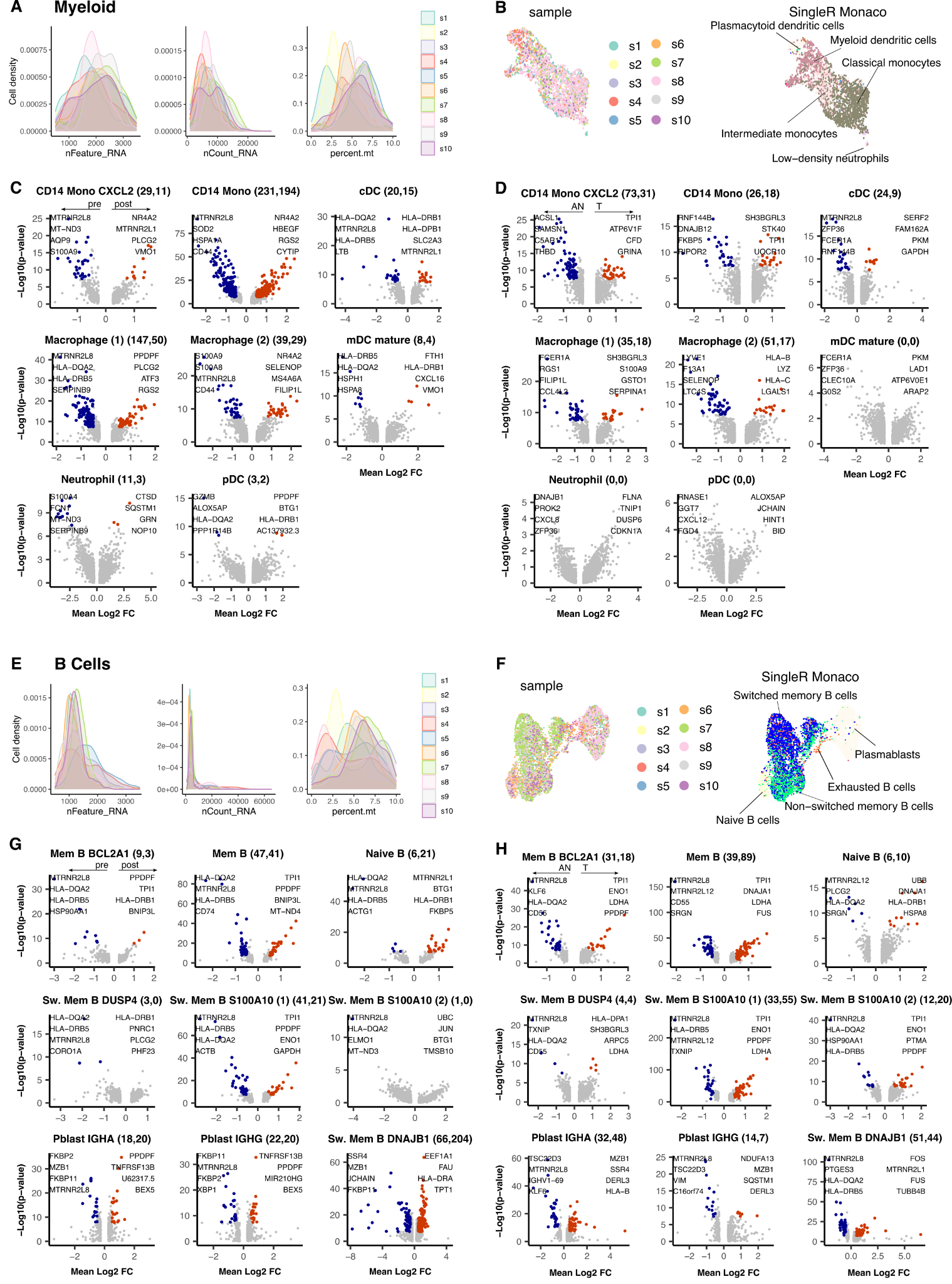


**Supplementary Figure S4. Quality metric distributions, SingleR annotations and differential expression analysis of Myeloid and B cells.** A, Samplewise distributions of Feature counts (nFeature_RNA), UMI counts (nCount_RNA) and % expression of Mitochondrial genes (percent.mt) for Myeloid (A) and B cells (E). B, Automated per-cell annotations of high level cell types using SingleR with the hpca coarse reference dataset for Myeloid (B) and B cells (F). C, Summary of genes identified as differentially expressed in post vs pre NACT EAC tumor sample data for Myeloid (C) and B cells (G). DEG count noted alongside cluster: (pre expressed, post expressed). D, Summary of genes identified as differentially expressed in EAC Tumor (T) vs Adjacent Normal (AN) sample data for Myeloid (D) and B cells (H). DEG count noted alongside cluster: (AN expressed, T expressed) Coloured points indicate DEGs (BH adjusted p<0.001 and absolute average log2FC > 0.5).


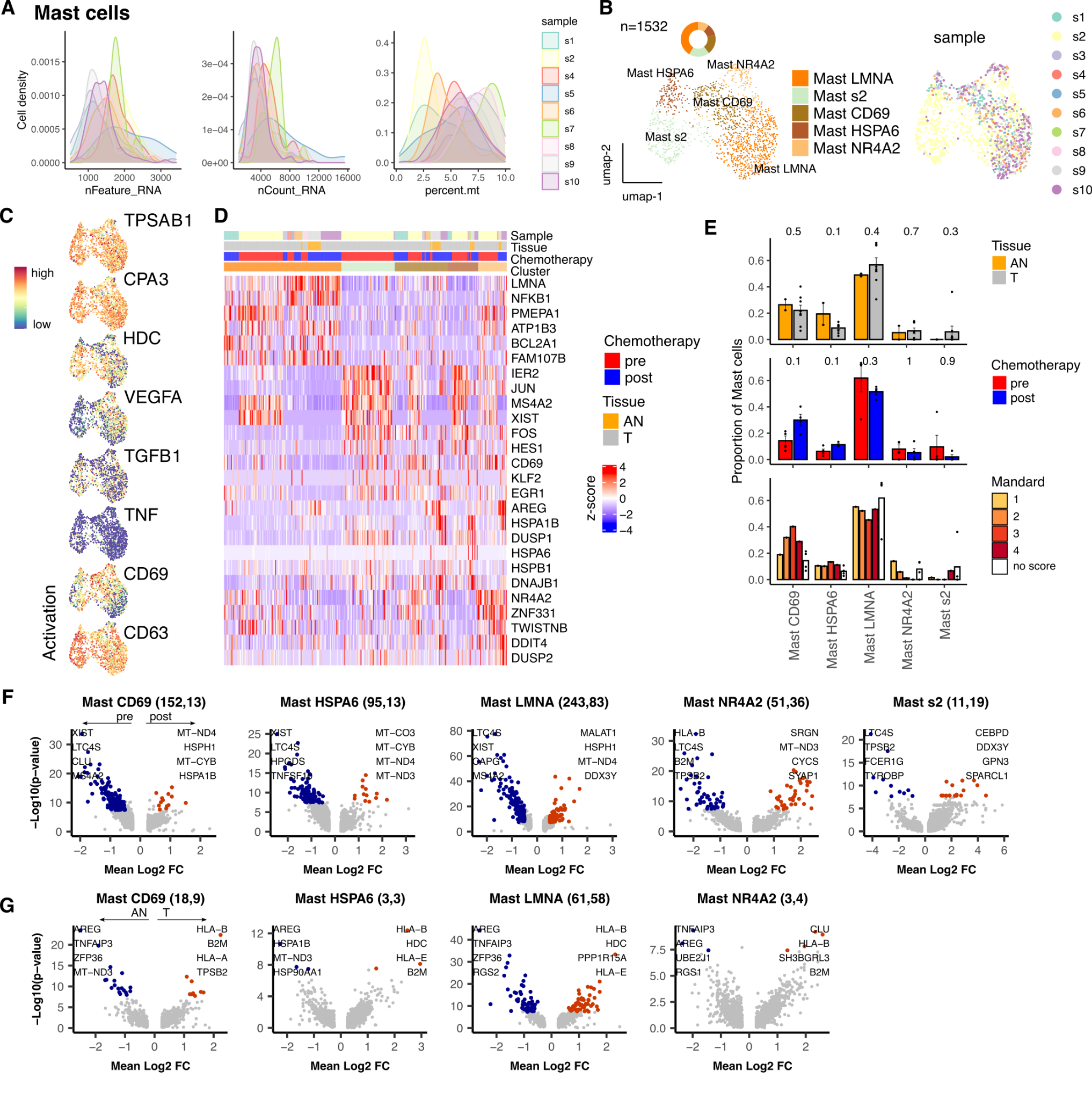


**Supplementary Figure S5. Quality metric distributions, SingleR annotations and differential expression analysis of Mast cells.** A, Samplewise distributions of Feature counts (nFeature_RNA), UMI counts (nCount_RNA) and % expression of Mitochondrial genes (percent.mt). B, Automated per-cell annotations of high level cell types using SingleR with the hpca coarse reference dataset. C, Summary of genes identified as differentially expressed in post vs pre NACT EAC tumor sample data for Mast cells (C) and Cycling cells (G,H). DEG count noted alongside cluster: (pre expressed, post expressed). D, Summary of genes identified as differentially expressed in EAC Tumor (T) vs Adjacent Normal (AN) sample data for Mast cells (D) and Cycling cells (I,J). DEG count noted alongside cluster: (AN expressed, T expressed) Coloured points indicate DEGs (BH adjusted p<0.001 and absolute average log2FC > 0.5).


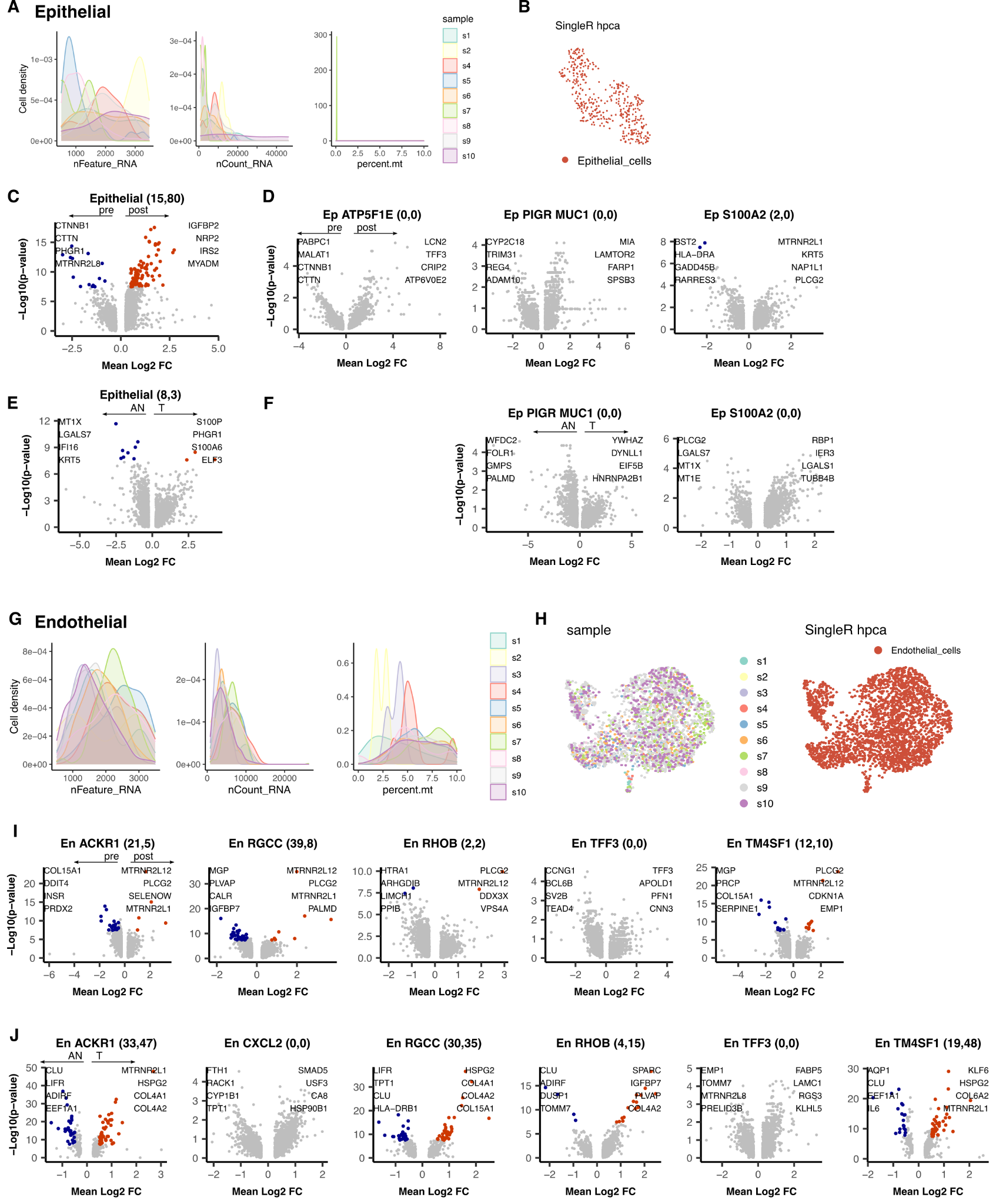


**Supplementary Figure S6. Quality metric distributions, SingleR annotations and differential expression analysis of Epithelial and Endothelial cells.** A, Samplewise distributions of Feature counts (nFeature_RNA), UMI counts (nCount_RNA) and % expression of Mitochondrial genes (percent.mt) for Epithelial (A) and Endothelial (G) cells. B, Automated per-cell annotations of high level cell types using SingleR with the hpca coarse reference dataset for Epithelial (B) and Endothelial (H) cells. C, Summary of genes identified as differentially expressed in post vs pre NACT EAC tumor sample data for all Epithelial cells (C) and within Epithelial sub-clusters (D). DEG count noted alongside cluster: (pre expressed, post expressed). E, Summary of genes identified as differentially expressed in EAC Tumor (T) vs Adjacent Normal (AN) sample data for all Epithelial cells (E) and within Epithelial sub-clusters (F). DEG count noted alongside cluster: (AN expressed, T expressed) Coloured points indicate DEGs (BH adjusted p<0.001 and absolute average log2FC > 0.5).


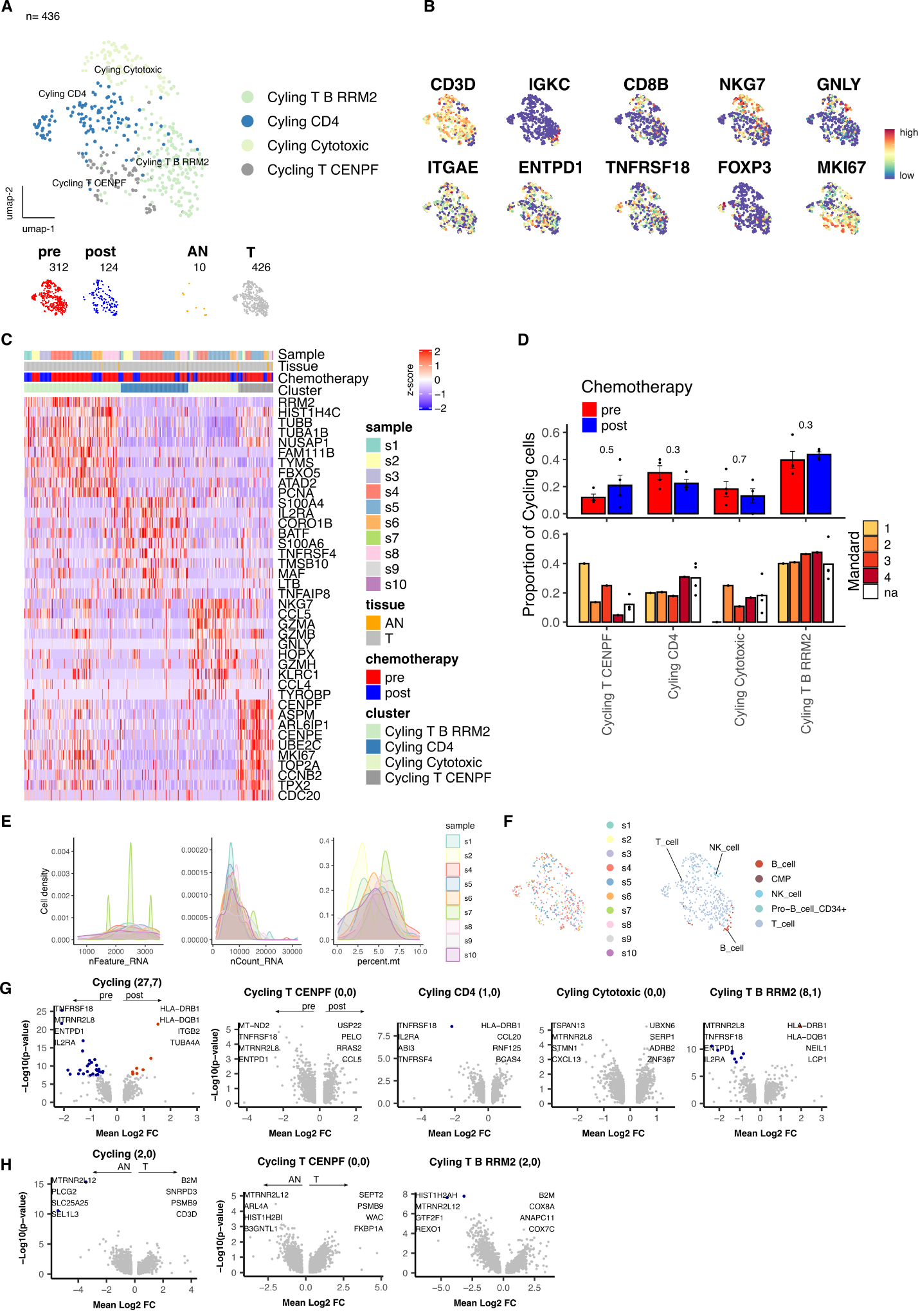


**Supplementary Figure S7. Quality metric distributions, SingleR annotations and differential expression analysis of Cycling cells.** A, UMAP embedding overlaid with cluster cell type annotations. B, UMAP embeddings overlaid with expression of canonical cell type markers of interest. C, Per-cell expression profile of top cluster marker genes. D, Comparison of pre vs post chemotherapy and Mandard score cluster proportions. Points represent within sample cluster proportion and p values determined by Mann-Whitney test. E, Samplewise distributions of Feature counts (nFeature_RNA), UMI counts (nCount_RNA) and % expression of Mitochondrial genes (percent.mt) for Epithelial (A) and Endothelial (G) cells. F, Automated per-cell annotations of cell types using SingleR with the hpca coarse reference dataset. G, Summary of genes identified as differentially expressed in post vs pre NACT EAC tumor sample data. DEG count noted alongside cluster: (pre expressed, post expressed). H, Summary of genes identified as differentially expressed in EAC Tumor (T) vs Adjacent Normal (AN) sample data. DEG count noted alongside cluster: (AN expressed, T expressed) Coloured points indicate DEGs (BH adjusted p<0.001 and absolute average log2FC > 0.5).
